# Comparative Effects of Using New Multi-Strain Synbiotics on Chicken Growth Performance, Hematology, Serum Biochemistry and Immunity

**DOI:** 10.3390/ani10091555

**Published:** 2020-09-02

**Authors:** Artur Żbikowski, Karol Pawłowski, Katarzyna Śliżewska, Beata Dolka, Joanna Nerc, Piotr Szeleszczuk

**Affiliations:** 1 Department of Pathology and Veterinary Diagnostics, Institute of Veterinary Medicine, Warsaw University of Life Sciences-SGGW, Nowoursynowska 159c St., 02-776 Warsaw, Poland; karol_pawlowski@sggw.edu.pl (K.P.); beata_dolka@sggw.edu.pl (B.D.); joanna_nerc@sggw.edu.pl (J.N.); piotr_szeleszczuk@sggw.edu.pl (P.S.); 2 Department of Biotechnology and Food Sciences, Institute of Fermentation Technology and Microbiology, Lodz University of Technology, Wolczanska 171/173, 90-924 Lodz, Poland; katarzyna.slizewska@p.lodz.pl

**Keywords:** broiler, gut microbiota, synbiotic, probiotic, immunity, antibiotic alternatives

## Abstract

**Simple Summary:**

Currently, there is an urgent need to decrease the use of antibiotics in poultry production. Long-term use of antibiotics in birds leads to the development of many drug-resistant microorganisms, has a negative impact on the environment and the antibiotic residues in poultry meat pose hazards to consumers’ health. Probiotics, prebiotics and synbiotics used in poultry are good alternatives for antibiotic and antibiotic growth stimulants. However, such additives must be adapted to the species, age and intended use of birds. Our newly developed synbiotic formulas which comprised three, four or five strains of *Lactobacillus* sp., as well as *S. cerevisiae* and inulin, were administered in feed for meat-type chickens throughout the 42-day experimental period. The results showed that our new synbiotics do not have any unfavorable influence on chicken health and may modulate the immune response and biochemical parameters. The results are very promising, and our synbiotics can be considered as products for commercial use in poultry. However, these findings should be confirmed in future field experiments.

**Abstract:**

In this study, the influence of new multi-strain synbiotics on chicken growth performance, hematology, serum biochemistry and immunity was explored. Each synbiotic preparation (A, B and C) comprised three, four or five strains of *Lactobacillus* sp., respectively, as well as *S. cerevisiae* and inulin. All strains used in the synbiotics originated from wild-type strains from animal farms in Poland. Six groups of chickens, ROSS 308 line, were fed with three different synbiotics at a dose of 0.5 g/1 kg of feed. Body weight, as well as the biochemical and hematological parameters of the animals in each study group, were determined on the 7th, 14th, 28th and 42nd day of life. Body weight on day 42 differed between groups and was the highest in control group. This group also had the highestfeed conversion ratio (FCR) value. All measured biochemical parameters were in the normal ranges for poultry; however, we observed a lower alkaline phosphatase (AP) concentration on day 7 in the groups fed with synbiotics, which correlated with a lower level of triglycerides in those groups. The aspartate transaminase (AST) concentration was significantly lower in all groups on day 42 in comparison with the control. On day 7, the control group showed the highest concentration of Ca, K and P. Other parameters did not differ significantly throughout the experiment. All groups showed a similar tendency of increase in the red blood cells (RBC) count according to the age of the birds. Every white blood cells (WBC) population showed differences in the proportions between T and B lymphocytes. The T cell and monocyte counts increased until day 28 in all groups. The results showed that our newly developed synbiotic formulas do not have any unfavorable influence on chicken health and may modulate immune response and biochemical parameters. However, this hypothesis needs to be evaluated in future experiments.

## 1. Introduction

Currently, there is an urgent need to decrease the use of antibiotics in animal production which requires finding new alternatives for animal health protection. Recently, much attention has been paid to the substitutes of antibiotic growth stimulants, such as probiotics, prebiotics, synbiotics, phytobiotics and acidifiers [1,2,3,4,5,6]. However, such additives must be adapted to the species, age and intended use of animals.

One of the criteria for the evaluation of the effect of such preparations are microbiological tests. Their results allow for determining the effect of growth stimulators used in feeding stuffs on the populations of microorganisms in the digestive tract of animals. According to Gedek and Kirchgessner [7], the gut microbiota is more heavily inhabited with bacteria of the genera *Lactobacillus, Bifidobacterium, Bacteroides* and *Eubacterium* (the most numerous flora) than with *Escherichia coli* (*E. coli*) and *Enterococcus* (less numerous flora). Other bacteria, including pathogenic ones such as *Staphylococcus, Proteus* and *Pseudomonas*, are much less abundant in the intestines (low flora). The ratio of 90:1:0.01 is considered the optimal ratio between the most numerous flora, less numerous flora and low flora, respectively. This condition is referred to as “eubiosis”. According to this concept, the role of growth-stimulating preparations is to support the main microbiota while inhibiting the development of other groups of microorganisms.

Antibiotic growth stimulants (AGSs) or antibiotic growth promoters (AGPs) have been widely used in poultry production to improve economic effects and to prevent infections caused by poultry pathogens dangerous for birds and consumers of poultry products [8]. Excessive use of AGSs (not only in animal production) has led to the development of drug-resistant bacteria, persistence of antibiotic residues tissues and disorders of the gastrointestinal tract [9]. For this reason, in January 2006, the European Union (EU) introduced a general prohibition of the use of antibiotics as growth promoters [10]. This decision contributed to the dynamic growth of interest in alternative methods to replace the need for antibiotic antimicrobials. The most common alternatives are probiotics, prebiotics, synbiotics, organic acids and herbal products. These product groups are widely accepted by consumers and considered “natural” and safe as feed additives.

The term “probiotic” was introduced into the literature by Lilly and Stillwell [11]. According to the currently most accepted definition, probiotics are single- or multi-component preparations containing live microorganisms that, when administered in appropriate numbers, bring health benefits to the host [12]. Probiotics improve the microbiological balance of intestines and strengthen the intestinal barrier against the influence of harmful factors [13]. Prebiotics are preparations containing indigestible nutrients that favorably affect the growth and/or activity of one or more intestinal bacterial species [14,15].

The combination of a probiotic and a prebiotic in one product is called a synbiotic. Synbiotics use a form of synergism, thanks to which probiotic bacteria find better conditions for development. Many studies have proven that synbiotics have a very diverse and beneficial effect on the birds receiving them. The range of this activity is very wide and includes ensuring intestinal integrity, limiting the growth of pathogenic bacteria, regulation of the immune system and absorption of nutrients [16]. Synbiotics stimulate the immune system very effectively, both local and peripheral [17,18,19]. The beneficial effects of synbiotics also include improving the quality of poultry products (meat and eggs) [20,21]. Probiotics have been widely recognized as safe products in human and veterinary medicine [22].

Theoretically, probiotics can be responsible for four types of side effects: systemic infections, harmful effects on the metabolism, excessive immune stimulation in susceptible individuals and transfer of resistance genes. A comprehensive report published in 2011 by the Agency for Research and Quality in the Health Service showed that clinical trials did not provide any evidence of increased risk for the use of probiotic preparations [23]. However, it should not be forgotten that, by interfering with the commensal microbiota, probiotics may cause opportunistic bacterial or fungal infections.

The present study aimed to evaluate the influence of the use of three new synbiotic preparations (A, B and C) on the health, immune response and biochemical parameters of chickens reared up to 42 days of age. The scope of observation included detailed clinical and laboratory tests. The aim was to be achieved by tracking the quantitative and qualitative changes in selected immune response elements (T cell and B cell subpopulations, monocytes), specific antibody levels and changes in blood morphology and biochemical indicators in chickens that received synbiotic preparations and two commercially available products in the feed. In addition, an assessment of the impact of these preparations on the health of birds during rearing was carried out based on the results of clinical observations, necropsy and laboratory tests. The economic aspects of production were also considered.

## 2. Materials and Methods

### 2.1. Birds, Diets and Experimental Design

The experimental group design is shown in Table 1.

The experiment was performed on a total of 144 chickens of both sexes, derived from a commercial broiler line (ROSS 308, Aviagen, France) obtained from a local commercial hatchery (ZWD Malec, Dębówka, Poland) and kept in an experimental broiler house (RZD Wilanow-Obory, Warsaw University of Life Sciences–SGGW, Warsaw, Poland). The birds were randomly allocated to six treatment groups in separate pens. Each group consisted of 24 birds. At every time point of sample collection, at 7, 14, 28 and 42 days of life, six birds (biological replicants) from each group were sacrificed. The broilers were kept on straw litter in a closed, ventilated broiler house equipped with bell drinkers and pan feeders to provide feed and water ad libitum throughout the 42-day experimental period. The temperature was maintained at 33 °C from day 0 to 7, after which it was gradually reduced to 24 °C at a rate of 3 °C per week and then maintained until the end of the experiment. Humidity in the broiler house was maintained at around 50%, and continuous lighting was provided throughout the experimental period.

Three different combinations of synbiotic formulas (marked as A, B, C) and two commercial probiotics: Bioplus^®^ YC, (Biochem, Karlsruhe, Germany) and Cylactin^®^ (DSM, Netherlands), were used. The choice of formulation of synbiotics was according to our previous experiments, largely described by Śliżewska et al. [24]. Each synbiotic preparation (A, B and C) comprised: 2 × 10^9^ CFU/g LAB (lactic acid bacteria) of *Lactobacillus* spp., 2 × 10^7^ CFU/g of *S. cerevisiae* yeast and 2% inulin (prebiotic), and was added into feed at a dose of 0.5 g/kg. All strains used in synbiotics A, B and C originated from wild-type strains isolated from animal farms in Poland. The strains have full probiotic documentation and are licensed [25,26,27,28,29,30]. The prebiotic and probiotic bacteria used in the synbiotic preparations were tested using procedures recommended by FAO/WHO and EFSA [12,31]. Bioplus^®^ YC and Cylactin^®^ were administered in the feed at a dose of 0.4 g/kg and 0.035 g/kg of feed, respectively, according to manufacturer indications. All complete dietetic compounds used in commercial feed for chickens were coccidiostat-free (Ekoplon, Grabki Duże, Poland). Detailed parameters of the feed used are presented in Table 2.

All experiments were conducted after obtaining the approval of III Local Ethical Commission for animal testing at Warsaw University of Life Sciences–SGGW, Poland, according to resolution No. 3/2015 of 22nd January 2015.

### 2.2. Determination of Chicken Production Parameters

Chickens were reared for 42 days and observed daily. The body weight of animals in each study group was determined at 7, 14, 28 and 42 days of life. Final production parameters, such as feed conversion ratio (FCR) and European production efficiency factor (EPEF), were determined according to the formulas: FCR = (feed consumption (kg))/(body weight gain (kg)), EPEF = (livability (%) × body weight (kg) × 100)/(age (days) × feed conversion ratio (kg)).

### 2.3. Determination of Blood Parameters

Blood samples (3 × 1 mL) were collected from the jugular vein of six birds in each group, at the following time points: 7, 14, 28 and 42 days of life. Blood was collected into two tubes coated with EDTAK3 for hematological and flow cytometry analysis, and into one tube without additives for serum intended for biochemical and serological tests. Red blood cell (RBC, 10^6^/µL) and white blood cell (WBC, 10^3^/µL) counts were determined in a Neubauer hematological chamber, with Natt and Herrick’s solution used as a solvent.

A chemistry analyzer (Miura One, I.S.E. S.r.l., Albuccione, Italy) was used for the determination of the following biochemical parameters of the serum: aspartate transaminase (AST, U/L), alanine transaminase (ALT, U/L), alkaline phosphatase (AP, U/L), glucose (mg/dL), uric acid (mg/dL), total protein (g/L), albumin (g/L), globulin (g/L) bilirubin (mg/mL), cholesterol (mg/dL), triglycerides (TG, mg/dL) and ions: calcium (Ca, mg/dL), phosphorus (P, mg/dL), potassium (K, mmol/L), sodium (Na, mmol/L). The activity of lysozyme (ng/mL) and selected acute phase proteins: ceruloplasmin (ng/mL), fibrinogen (ng/mL), haptoglobin (ng/mL), IF-gamma (pg/mL) and levels of IgY (ng/mL), IgM (ng/mL), IgA (ng/mL) were determined using ELISA and commercial kits, according to the manufacturer’s protocols (Finetest, Hubei, China). Peripheral blood T and B lymphocytes and monocytes were isolated using low-speed centrifugation and Histopaque^®^1077 (Sigma-Aldrich, St. Louis, MO, USA) density gradient separation. Specific monoclonal antibodies, mouse anti-chicken CD3+ and CD4+ (fluorescein isothiocyanate, FITC staining), CD8+, Bu-1+ and monocyte/macrophage+ (phycoerythrin, PE staining) (SouthernBiotech, Birmingham, AL, USA), were used. Fluorescence intensities were measured on a BD FACSAriaTM flow cytometer, and the results were analyzed using BD FlowJo^®^ software (Ashland, OR, USA).

### 2.4. Statistical Analysis

The results were analyzed using the appropriate statistical tests (ANOVA, Tukey’s test) and GraphPad Prism 7 software (San Diego, CA, USA) and presented as mean ± standard deviation (SD). Significant differences were accepted at *p* ≤ 0.05 with the rate of acceptance at 95%.

## 3. Results

### 3.1. The Effect of Dietary Treatment on Production Parameters

Basic growth performance parameters, such as body weight, average cumulative feed consumption, FCR and EPEF, are shown in Table 3.

On day 7, body weight differed among the groups and varied from 107.53 ± 11.82 g (group B) to 137.93 ± 8.48 g in group K (without additives). The same tendency was noticed regarding the average cumulative feed consumption and EPEF. No differences in these parameters were found among the groups at days 14 and 28, except FCR at day 14 when it was higher in group K compared with the other groups. At day 42, the FCR value, which is based on body weight and feed consumption, was the highest in group K. It was in line with the significant differences in body weight found between the groups: the highest was in group K, 2235.00 ± 67.71 g, and decreased to 1944.00 ± 66.28 g in groups D, E, C, B and A. EPEF ranged in that period from 280.96 ± 11.52 to 306.70 ± 2.37 in the experimental groups, and was 309.90 ± 16.67 in the control group.

### 3.2. The Effect of Dietary Treatment on Chicken Biochemical Parameters

To better understand the interactions of feed additives, we evaluated the biochemical blood parameters throughout the experiment. All measured parameters were in the normal ranges for poultry. However, we noticed some differences among groups. At day 7, the AP concentration was significantly lower in groups A, B and C in comparison with groups E and K. Additionally, groups A and B had lower AP values than group D. At day 14, we noticed an increase in AP in groups A, B and C and a decrease in other groups. The AP concentration decreased from day 28 until the end of the experiment in all groups, with no significant differences between the groups observed. No changes regarding ALT were observed throughout the experiment, while the AST concentration showed no changes except at the last time point, when it was significantly different in all groups, with that in group K being the highest (834.6 ± 123.7 U/L) (Table 4).

Other measured biochemical parameters showed significant differences at day 7, with triglyceride concentrations being much higher in groups E and K than in groups A, B, C and D. From this time point, their levels decreased gradually in all groups. On day 14, the only parameter for which differences were observed was bilirubin concentration. It was the highest in group D, and the difference was significant in comparison with other groups receiving food additives. The bilirubin concentration was also significantly higher in group D compared with groups B, C and E at day 42. The greatest number of differences was found at day 28. The glucose concentration was higher in groups A and E than in groups B, C and D. Uric acid at a concentration of 9.9 ± 6.0 mg/dL in group K was significantly higher than in groups A, D and E. Additionally, group C had a significantly higher uric acid concentration than group A (Table 5).

Group C had the highest total protein concentration and the difference was significant compared with groups E and K at day 28. Interestingly, the concentration of albumin observed at this day was the lowest in group A and significantly different from all other groups (Table 6).

Despite some differences between the groups at different time points, we observed a similar evolution of the parameters measured in each group over time.

To gain a general view on the health status and physiology of the experimental chickens, we measured the concentrations of the main macroelements in serum (Table 7). The profile of calcium fluctuations during the experiment was similar in all groups, with some differences noticed between the groups at days 7 and 14. At the first time point, groups A, B and C exhibited a lower concentration of calcium than groups E and K. Moreover, group C had a significantly lower calcium concentration than groups A and D. At day 7, we observed a lower concentration of phosphorus in group B in comparison with groups E and K. Furthermore, at day 7, groups A, B and C exhibited a lower concentration of potassium than group D. Additionally, groups B and C had a lower potassium level than groups E and K. In the case of both measured macroelements, some differences were observed at day 28. Group D presented a higher phosphorus concentration than group K, and groups C and D showed a higher concentration of potassium than groups E and K. The concentration of sodium was quite stable throughout the experiment, and the only significant differences were found at day 7 between groups B and D (151.0 ± 7.1 mmol/L and 137.8 ± 3.7 mmol/L, respectively), and at day 42 when group A showed a significantly lower sodium concentration (131.2 ± 24.3 mmol/L) compared with all other groups (Table 7).

### 3.3. The Effect of Dietary Treatment on Chicken Hematology

**Figure 1:**
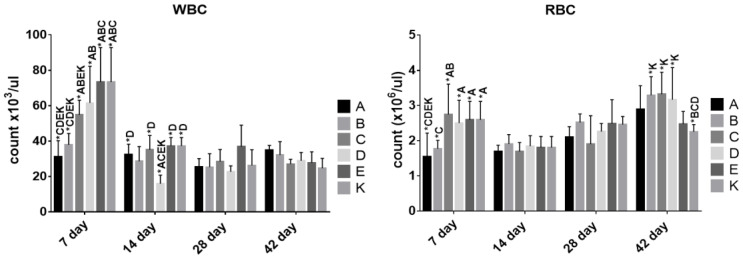
Average red blood cells (RBC) and white blood cells (WBC) count in the blood of the experimental chickens. *—statistically significant differences between groups, *p* ≤ 0.05.

The influence of feed additives was also evaluated by analyzing the blood of the chickens (Figure 1).

The average number of RBCs varied at day 7 and was the highest in group C and the lowest in group A. The latter group differed significantly from groups C, D, E and K. Despite the differences observed on day 7, we did not notice them at days 14 and 28. The differences appeared again at day 42 when group K showed a significantly lower RBC count then groups B, C and D, but the groups receiving food additives did not show differences among them.

A similar tendency was observed regarding WBC count. Differences appeared at day 7 when the cell count was significantly higher in groups K and E in comparison with groups A, B and C. Groups D and C had a higher cell count than groups A and B, which had the lowest cell counts at that sampling time point. The number of WBCs decreased significantly in all groups at the subsequent time points and became similar between the groups. Only group D had a significantly lower cell count compared with groups A, C, E and K on day 14.

### 3.4. The Effect of Dietary Treatment on Chicken Lymphocyte and Monocyte Populations

**Figure 2:**
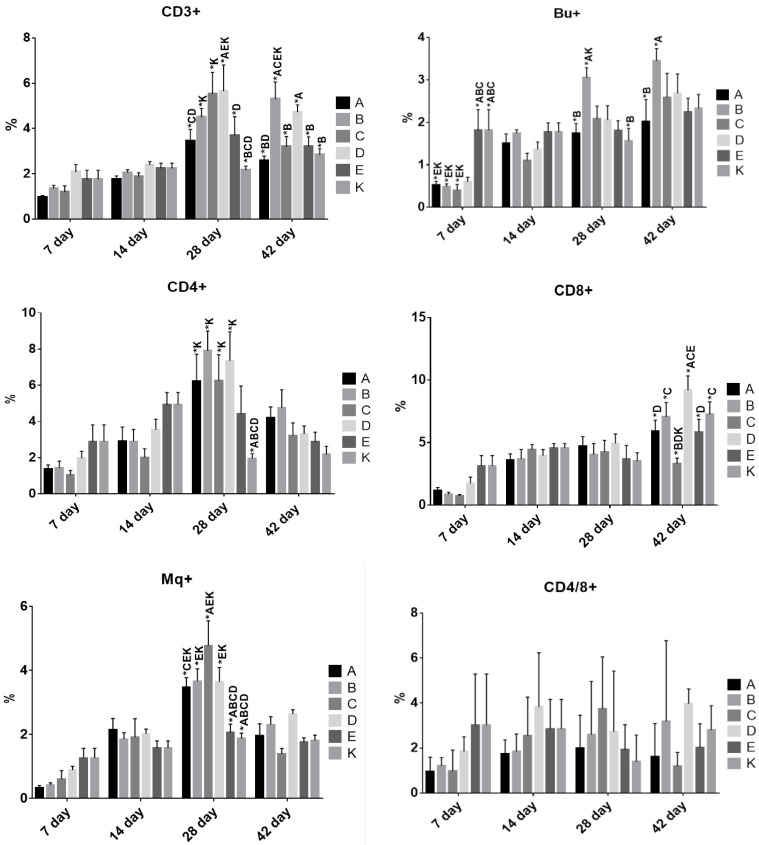
Flow cytometry analysis of the percentage of T lymphocytes with CD3^+^, CD4^+^, CD8^+^, CD4^+^/8^+^ receptors, B lymphocytes (Bu-1^+^) and monocytes (Mq^+^) in the blood of the experimental chickens. *—statistically significant differences between groups, *p* ≤ 0.05.

As the total WBC count was a general parameter, flow cytometry analysis was performed to evaluate each WBC population (Figure 2).

In the first panel, we observed an elevation of the percentage of CD3+ and Bu-1+ cells (representing T and B cells, respectively) in all groups and sampling time points. The tendency in both cases was that T cells rose from day 28 in all groups, and a similar but less pronounced change over time was observed for B cells. CD3+ cells did not differ among the groups at days 7 and 14. Their percentage rose on day 28 in all groups and was higher in group D in comparison with groups A, E and K. A significantly higher percentage of these cells was also observed in groups B and C compared with group K. The value for group A was lower than for groups C and D on day 28. At day 42, the situation changed, and the highest percentage of T cells was observed in group B, with a significant difference compared with groups A, C, E and K. Moreover, the value for group A was significantly lower than for group D.

The second panel was designed to demonstrate changes within the CD4+ (T helper cells) and CD8+ (T cytotoxic cells) populations. Observations of changes in the average percentage of CD4+ cells in time revealed a tendency to increase in all groups until day 28 and to decrease at day 42. Only group K seemed to remain at a low level throughout the experiment. The CD8+ population was at a relatively low level in all experimental groups, with significant differences among the groups noticed at day 42. Group C had a significantly lower percentage of CD8+ cells than groups B, D and K. Group D had a higher percentage in comparison with groups A, C and E.

The same tendency to increase until day 28, as for the other T cell populations, was observed in the case of Mq+ cells. At that time point, groups E and K had the lowest percentage of these cells which was significantly different from that in groups A, B, C and D. Additionally, group C had the highest percentage of Mq+ which was significantly different from that in group A. At day 42, Mq+ decreased in all experimental groups.

### 3.5. The Effect of Dietary Treatment on Chicken Acute Phase Proteins and Immunoglobulins

**Figure 3:**
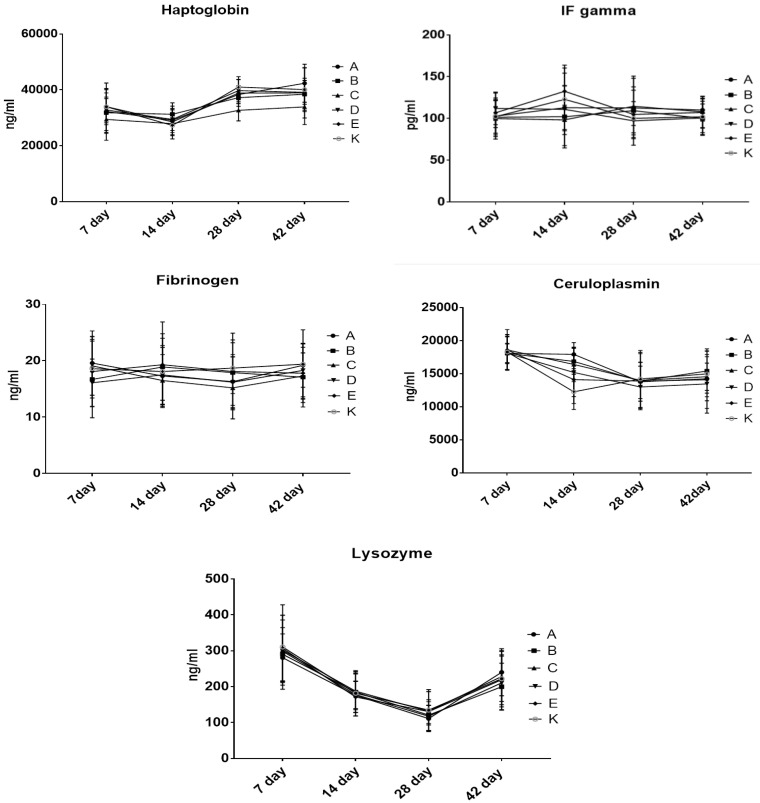
Levels of lysozyme and selected acute phase proteins in the chicken blood.

**Figure 4:**
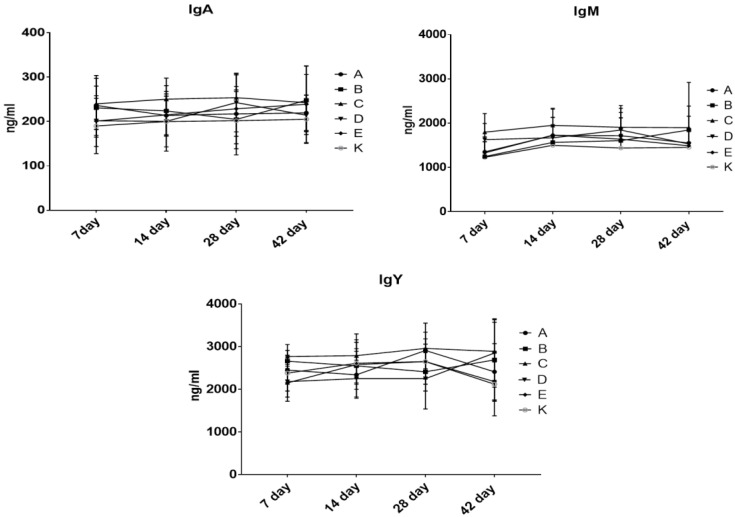
Levels of IgY, IgM and IgA in the chicken blood.

To evaluate the influence of the tested substances on the immune system function, the levels of lysozyme, acute phase proteins (Figure 3) and immunoglobulins (Figure 4) were measured in the chicken serum. The levels of the evaluated parameters did not differ among the groups at each time point and did not change significantly over time.

## 4. Discussion

### 4.1. Growth Performance of Broilers

Production parameters are often used for the evaluation of the influence of synbiotics and probiotics on the health of broiler chickens. Many experimental models have shown that these nutritional supplements usually improve the performance of broiler chickens, but the results depend on the type of synbiotic used [1,10,11,13,15,16,18,21,32,33,34,35,36,37,38,39,40,41,42,43]. Usually, these parameters are measured at the end of the production cycle but, to improve the quality of analysis, we decided to measure them at several time points. We observed that the body weight of chickens was lower in the group fed with synbiotics and Cylactin^®^ on days 7 and 42 in comparison with the control. However, FCR was lower in groups A, B and C on day 42. This parameter clearly indicates that the birds fed with synbiotics consumed less feed per 1 kg of body weight gain than the birds from the control group, which is positive and desirable from an economical point of view. The differences in body weight may be due to an uneven number of birds of both sexes in different groups. Birds for testing were chosen randomly within each group, which is why FCR seems to be a parameter that better reflects reality. Moreover, we noticed lower FCR values in all groups receiving synbiotics and probiotics, which is a sign of better production efficiency and may confirm a beneficial influence of these additives on chicken performance.

The performance of broiler birds was also evaluated in terms of the European production efficiency factor (EPEF), which includes daily weight gain and survival percentage. Higher values of EPEF indicate that the body weight gain in birds is uniform, and the flock is in good health [44]. The EPEF value in group A at 42 days of age was significantly lower, but there were no statistically significant differences between the results from other groups in comparison with the control group.

### 4.2. Serum Biochemistry and Immunity

Many authors have studied the effect of administering synbiotics or probiotics on the activity of selected enzymes in blood serum [18,32,37,38,40,42,45].

In our experiment, all measured biochemical parameters were in the normal ranges for poultry, but some differences were noticed between the groups. Alkaline phosphatase is derived from the bile duct epithelial cells, intestinal mucosa and kidneys. The AP activity can be high in young birds during growth and decreases with age. We observed a lower AP concentration at day 7 in the groups fed with synbiotics, which correlates with a lower level of triglycerides in those groups. It may be an effect of decreasing lipogenesis in the liver. Similar results were observed by Alimohamadi et al. [38]. They found a similar decrease in the blood triglyceride concentration in chickens receiving a prebiotic (Fermacto) and a probiotic (Primalac). There were no changes regarding ALT during the whole experiment, and only the AST concentration in group K differed significantly from that in all other groups (the highest value). AST is involved in the transformation of proteins, but there was no correlation with the level of albumin, globulin or total protein in the chicken blood during the experiment. In chickens, the major bile pigment biliverdin may be converted to bilirubin by bacteria or non-specific reducing enzymes [46], which may explain the higher bilirubin concentration at days 14 and 42 in chickens fed with a commercial probiotic (Bioplus^®^ YC).

Uric acid is the main product of protein nitrogen and purine metabolism in poultry. Approximately 90% of uric acid is secreted by the proximal convoluted tubules in healthy broilers [47]. The relatively low level of uric acid allowed us to exclude kidney diseases in the birds from all groups, and there were no significant differences between the groups until 28 days of life. At that time point, we observed higher values in birds from the control group which may be a sign of a beneficial effect of feed additives on the functioning of chicken kidneys. Interestingly, at the same time, we noticed a higher level of total protein, which can be attributed to higher albumin values in birds fed with synbiotic C. High values usually reflect a high protein metabolism rate but it was not correlated with a higher body weight in the chickens from that group at that time point. Sodium, potassium, calcium and phosphorus, present in blood and cellular fluids in the form of electrolytes, affect the osmotic pressure and acid–base balance in the body. Further evaluation of ions in serum showed that the Ca and P values were within their normal ranges for chickens and varied depending on bird age. However, some differences were noticed among the groups at days 7 and 14, for example, group C had the lowest Ca concentration at day 7 and one of the highest at day 14. Moreover, there were no statistical differences in the Ca:P ratio throughout the experiment. Similarly, the concentration of Na was quite stable during the experiment, and the values were within their normal ranges for chickens. A different situation was observed in the evaluation of the K levels between time points. All groups exhibited fluctuations in this ion level. Lower values were found at days 7 and 28, and higher at days 14 and 42. Only small differences in biochemical parameters were observed between groups, and no unfavorable effect of the feed additives used was found.

The effect of using different synbiotic formulas or probiotics on chicken immunity has also been evaluated by other authors using different hematological tests [1,34,38,39,45,48,49,50]. Probiotic bacteria as a nutritional component could stimulate the local immune system in the gut; however, their systemic effects would be observable in blood. It has been reported that different LAB strains have different immunomodulatory effects, thus identical effects cannot be expected when different bacterial strains with probiotic potential are used [51]. The first parameter evaluated was the RBC average count. All groups showed a similar tendency of increase in the RBC count according to the age of birds. Indeed, only the groups receiving a synbiotic (A, B and C) showed significant differences between days 7 and 14, 14 and 28, 28 and 42. It may suggest a better absorption of nutrients necessary for erythropoiesis. The differences in the WBC average count which appeared on day 7 between the groups may indicate that the intestinal flora of the chicks up to the 7th day of life stimulated the intestinal immune system and, as a result, led to transient leukocytosis. A similar effect was also observed by Hanamanta et al. [49]. Each WBC population evaluated using flow cytometry showed differences in the proportions between T and B lymphocytes. The tendency was that levels of T cells and monocytes rose until day 28 in all groups, and a similar but less pronounced change over time was observed for B cells. Observing the T cell population, we noticed the tendency of the average percentage of CD4+ cells to increase until day 28 and to decrease at day 42. These variable changes may be due to the activation of CD4+ T cells (a few days after a challenge in the mucosal areas by the synbiotics and the probiotic) which reach their maximum levels within 14 days. Subsequently, they may be recruited to peripheral blood. The CD8+ population was at a relatively low level in all experimental groups, and only at day 42 did we observe a general increase in these cells and some differences among the groups. The different profiles in group C and a significantly lower level of CD8+ at day 42 can be explained by a low level of stimulation of cell-mediated immunity.

The levels of lysozyme, acute phase proteins (Figure 3) and immunoglobulins (Figure 4) measured in the chicken serum did not differ among the groups at each time point and did not change significantly over time. In addition, other researchers have found that probiotic bacteria do not alter the number of serum immunoglobulins Y, IgM or IgA [18,52,53].

## 5. Conclusions

The results showed that our experimental formulas of newly developed synbiotics do not have any unfavorable influence on chicken health and may modulate immune response and biochemical parameters. However, these findings should be confirmed in future field studies.

## Figures and Tables

**Table 1:** Experimental group design.

Experimental Groups		Composition
**Synbiotic preparation**	**A**	*Lactobacillus plantarum* (ŁOCK 0860)
		*Lactobacillus reuteri* (ŁOCK 1092)
		*Lactobacillus pentosus* (ŁOCK 1094)
		*Saccharomyces cerevisiae* (ŁOCK 0119)
	**B**	*Lactobacillus plantarum* (ŁOCK 0860)
		*Lactobacillus reuteri* (ŁOCK 1092)
		*Lactobacillus pentosus* (ŁOCK 1094)
		*Lactobacillus rhamnosus* (ŁOCK 1087)
		*Saccharomyces cerevisiae* (ŁOCK 0119)
	**C**	*Lactobacillus plantarum* (ŁOCK 0860)
		*Lactobacillus reuteri* (ŁOCK 1092)
		*Lactobacillus pentosus* (ŁOCK 1094)
		*Lactobacillus rhamnosus* (ŁOCK 1087)
		*Lactobacillus paracasei* (ŁOCK 1091)
		*Saccharomyces cerevisiae* (ŁOCK 0119)
Probiotics	**D**	Bioplus^®^ YC:
		*Bacillus licheniformis* (DSM 5749)
		*Bacillus subtilis* (DSM 5750)
	**E**	Cylactin^®^:
		*Enterococcus faecium* NCIMB 10415 (SF68), LBC ME10, DSM
Control	**K**	Without additives

**Table 2:** The composition of the complete dietetic commercial mixtures of feed used in the experiment.

	Type of Feed		
Name	Broiler Starter Prestige	Broiler Grower Prestige	Broiler Finisher Prestige
Composition	wheat, corn, post-extraction soya meal *, hemoglobin (from swine blood), rapeseed cake, soybean oil *, calcium carbonate, palm oil derived fatty acids, monocalcium phosphate, sodium chloride	wheat, corn, post-extraction soya meal *, hemoglobin (from swine blood), rapeseed cake, post-extraction sunflower meal, palm oil derived fatty acids, soybean oil *, calcium carbonate, swine fat, monocalcium phosphate, sodium chloride	wheat, corn, post-extraction soya meal * rapeseed cake, soybean oil *, calcium carbonate, monocalcium phosphate, sodium chloride
**Component amount in 1 kg of feed**			
Total proteins (Kjeldahl method)	225 g	205 g	187.5 g
Oil and crude fats	48 g	55 g	75 g
Fiber	27 g	30 g	33 g
Lysine	13.6 g	12.9 g	12.2 g
Methionine	6 g	5.8 g	5.6 g
Calcium	8 g	6.2 g	4.9 g
Phosphorus	6 g	5.2 g	4.6 g
Sodium	1.5 g	1.5 g	1.4 g
Ash	55 g	47 g	40 g
**Dietetic additives in 1 kg of feed**			
Vitamin A (3a672a)	10,000 IU		
Vitamin D3 (E671)	5000 IU		
**Vitamin E (dl-**α**-tocopherol)**	75 mg		
Fe (iron sulphate, E1)	40 mg		
J (potassium iodide, 3b201)	1.25 mg		
Cu (copper sulphate, E4)	16 mg		
Mn (manganese oxide, E5)	120 mg		
Zn (zinc oxide, E6)	100 mg		
Se (sodium selenite, E8)	0.3 mg		
**Zootechnics additives in 1 kg of feed**			
**6-phytase (EC 3.1.3.26) 500 FTU/g, 4a19**	1000 FTU	1000 FTU	-
**6-phytase (EC 3.1.26) 2500 OTU/g, 4a16**	-	-	250 OTU/g
**Endo-1,4-**β**-xylanase (EC 3.2.1.8), 12,500 VU/mL, 4a22**	1250 VU	1250 VU	-
**Endo-1,4-**β**-xylanase (EC 3.2.1.8), 30,000 EPU/g, 4a1617**	-	-	1500 EPU
**Endo-1,3 (4)-**β**-gluconate (EC 3.2.1.6), 8600 VU/mL, 4a22**	860 VU	860 VU	-
**Serine protease (EC 3.4.21), 75,000 PROT/g 4a13**	15000 PROT	-	-

**EPU**—one endo-1,4-β-xylanase unit is the amount of enzyme which releases 0.0083 μmol of reducing sugars (xylose equivalent) per minute from oat spelt xylan at pH 4.7 and 50 °C; **FTU**—one 6-phytase unit is the amount of enzyme which releases 1 µmol of inorganic phosphate from sodium phytate in one minute at 37 °C and pH 5.5; **PROT**—one protease unit is the amount of enzyme which releases 1 µmol of *p*-nitroaniline from 1 mM substrate (Suc-Ala-AlaPro-Phe-pNA) in one minute at pH 9.0 and temperature 37 °C; **VU**—one endo-1,3(4)-β-gluconate unit is the amount of enzyme which hydrolyzes substrate (β-glucan of barley and arabinoxylan of wheat, respectively), while reducing viscosity of the solution, so that there is a change in relative fluidity of 1 (dimensionless unit) in one minute at 30 °C and pH 5.5; *—produced from GMO soybeans (MON 40-3-2).

**Table 3:** Production parameters of chickens fed with the synbiotic- or probiotic-supplemented feed from the following sampling time points: 7, 14, 28 and 42 days of chicken life. *—statistically significant differences between groups, *p* ≤ 0.05.

Age of Birds	Feed Additives	Body Weight (Mean ± SD) (g)	Average Cumulative Feed Consumption (g)	Feed Converse Ratio (FCR)	European Production Efficiency Factor (EPEF)
**7 days**	Synbiotic A	110.71 ± 12.02 *^DK^	96.00 ± 9.78 *^DEK^	0.87 ± 0.03	182.66 ± 23.29 *^EK^
	Synbiotic B	107.53 ± 11.82 *^DEK^	95.00 ± 9.78 *^DEK^	0.89 ± 0.10	175.99 ± 39.36 *^EK^
	Synbiotic C	108.32 ± 7.81 *^DEK^	95.93 ± 2.52 *^DEK^	0.89 ± 0.05	175.33 ± 23.22 *^EK^
	Bioplus^®^ YC (D)	134.43 ± 7.53 *^ABCE^	116.00 ± 6.75 *^ABCE^	0.86 ± 0.05	223.21 ± 22.03
	Cylactin^®^ (E)	120.66 ± 10.35 *^BCDK^	105.00 ± 7.38 *^ABCDK^	0.87 ± 0.03	198.23 ± 20.97 *^ABCK^
	Without (K)	137.93 ± 8.48 *^ABCE^	117.00 ± 6 *^ABCE^	0.85 ± 0.02	232.41 ± 18.04 *^ABC^
**14 days**	Synbiotic A	288.65 ± 23.01	323.17 ± 23.44	1.12 ± 0.02	182.19 ± 16.27
	Synbiotic B	298.69 ± 17.19	330.67 ± 14.68	1.11 ± 0.05 *^K^	193.01 ± 17.77
	Synbiotic C	305.60 ± 26.73	334.33 ± 16.69	1.10 ± 0.05 *^K^	199.86 ± 25.85
	Bioplus^®^ YC (D)	308.42 ± 23.60	338.33 ± 19.81	1.10 ± 0.04	198.85 ± 20.55
	Cylactin^®^ (E)	299.80 ± 38.32	334.83 ± 36.84	1.12 ± 0.02 *^K^	189.71 ± 27.80
	Without (K)	302.90 ± 17.04	361.67 ± 30.96	1.19 ± 0.08 *^BCE^	179.94 ± 16.44
**28 days**	Synbiotic A	1121.33 ± 130.45	1608.5 ± 234.84	1.43 ± 0.07	279.78 ± 28.53
	Synbiotic B	1087.50 ± 64.17	1510.17 ± 130.63	1.39 ± 0.06	276.87 ± 17.13
	Synbiotic C	1096.33 ± 116.77	1493.5 ± 139.34	1.36 ± 0.04	284.23 ± 35.70
	Bioplus^®^ YC (D)	1180.17 ± 79.61	1643.29 ± 88.55	1.39 ± 0.04	307.75 ± 26.60
	Cylactin^®^ (E)	1130.00 ± 122.76	1568.33 ± 143.08	1.39 ± 0.05	290.06 ± 37.82
	Without (K)	1113.67 ± 134.02	1540.14 ± 202.76	1.39 ± 0.05	280.04 ± 29.93
**42 days**	Synbiotic A	1944.00 ± 66.28 *^BCDEK^	3168.50 ± 114.51 *^BCDEK^	1.63 ± 0.03 *^K^	280.96 ± 11.52 *^BCK^
	Synbiotic B	2095.33 ± 18.14 *^AK^	3352.17 ± 45.67 *^ADEK^	1.60 ± 0.01 *^DEK^	304.43 ± 2.52 *^A^
	Synbiotic C	2096.17 ± 12.43 *^AK^	3374.00 ± 26.5 *^ADEK^	1.61 ± 0.01 *^DEK^	306.70 ± 2.37 *^A^
	Bioplus^®^ YC (D)	2176.50 ± 56.95 *^AK^	3677.33 ± 87.71 *^ABC^	1.69 ± 0.05 *^BC^	299.62 ± 14.79
	Cylactin^®^ (E)	2119.67 ± 39.51 *^A^	3561.50 ± 133.74 *^ABCK^	1.68 ± 0.05 *^BC^	297.31 ± 9.48
	Without (K)	2235.00 ± 67.71 *^ABCD^	3798.00 ± 43.82 *^ABCE^	1.70 ± 0.04 *^ABC^	309.90 ± 16.67 *^A^

**Table 4:** Activity of the tested enzymes (aspartate transaminase (AST), alanine transaminase (ALT), alkaline phosphatase (AP) in the blood serum of the experimental chickens. *—statistically significant differences between groups, *p* ≤ 0.05.

Age of Birds	Feed Additives	AST (U/L)	ALT (U/L)	AP (U/L)
**7 days**	Synbiotic A	261.6 ± 42.7	9.8 ± 5.2	2839.1 ± 397.6 *^DEK^
	Synbiotic B	228.1 ± 33.3	8.9 ± 3.3	3051.0 ± 572.0 *^DEK^
	Synbiotic C	263.3 ± 63.9	6.2 ± 3.7	3556.2 ± 1345.3 *^EK^
	Bioplus^®^ YC (D)	248.7 ± 24.3	6.0 ± 1.9	4763.9 ± 708.7 *^AB^
	Cylactin^®^ (E)	239.6 ± 42.7	5.7 ± 2.2	5032,6 ± 104.1 *^ABC^
	Without (K)	220.6 ± 21.1	6.0 ± 3.0	4957.2 ± 165.3 *^ABC^
**14 days**	Synbiotic A	237.3 ± 22.7	6.9 ± 4.2	4718.0 ± 627.3
	Synbiotic B	228.3 ± 16.9	3.7 ± 3.3	4266.4 ± 535.6
	Synbiotic C	234.1 ± 15.1	6.6 ± 4.7	4021.2 ± 819.9
	Bioplus^®^ YC (D)	247.0 ± 31.1	6.1 ± 4.3	3883.8 ± 1272.6
	Cylactin^®^ (E)	225.6 ± 15.7	8.7 ± 3.2	4684.9 ± 616.6
	Without (K)	224.8 ± 9.7	9.1 ± 4.6	4602.5 ± 1061.2
**28 days**	Synbiotic A	264.1 ± 42.4	8.6 ± 6.6	2067.8 ± 506.9
	Synbiotic B	304.1 ± 63.8	7.3 ± 5.2	1567.6 ± 308.0
	Synbiotic C	307.5 ± 73.4	7.6 ± 4.2	2462.9 ± 339.8
	Bioplus^®^ YC (D)	277.9 ± 33.9	8.3 ± 4.0	2025.1 ± 799.8
	Cylactin^®^ (E)	253.3 ± 46.8	5.4 ± 3.3	1874.0 ± 710.1
	Without (K)	279.0 ± 53.6	8.8 ± 2.6	2250.3 ± 1686.2
**42 days**	Synbiotic A	299.8 ± 107.4 *^K^	3.9 ± 1.6	1255.7 ± 286.6
	Synbiotic B	384.2 ± 31.1 *^K^	4.8 ± 3.6	940.2 ± 264.2
	Synbiotic C	376.6 ± 63.5 *^K^	6.0 ± 2.8	792.1 ± 276.8
	Bioplus^®^ YC (D)	365.2 ± 91.5 *^K^	7.4 ± 4.5	1774.9 ± 320.8
	Cylactin^®^ (E)	402.4 ± 20.0 *^K^	8.0 ± 4.8	1057.0 ± 326.8
	Without (K)	834.6 ± 123.7 *^ABCDE^	5.0 ± 2.0	1728.0 ± 303.7

**Table 5:** Concentrations of the tested parameters in the blood serum of the experimental chickens. *—statistically significant differences between groups, *p* ≤ 0.05.

Age of Birds	Feed Additives	Glucose (mg/dL)	Uric Acid (mg/dL)	Bilirubin (mg/dL)	Cholesterol (mg/dL)	Triglycerides (mg/dL)
**7 days**	Synbiotic A	270.8 ± 31.9 *^D^	6.3 ± 1.1	0.3 ± 0.3	211.4 ± 20.4	80.8 ± 39.1 *^EK^
	Synbiotic B	250.3 ± 26.8	7.9 ± 2.8	0.3 ± 0.1	193.0 ± 9.9	82.5 ± 26.0 *^EK^
	Synbiotic C	244.9 ± 14.0	5.4 ± 2.1	0.4 ± 0.3	193.8 ± 11.7	44.2 ± 11.1 *^EK^
	Bioplus^®^ YC (D)	214.1 ± 17.6 *^A^	5.7 ± 1.1	0.3 ± 0.0	175.2 ± 21.3	68.2 ± 32.2 *^EK^
	Cylactin^®^ (E)	239.1 ± 28.2	6.0 ± 1.5	0.3 ± 0.1	181.4 ± 26.9	132.9 ± 32.9 *^ABCD^
	Without (K)	228.3 ± 23.4	5.9 ± 1.2	0.3 ± 0.1	178.2 ± 25.2	135.0 ± 28.5 *^ABCD^
**14 days**	Synbiotic A	225.9 ± 12.5	5.6 ± 0.9	0.2 ± 0.1 *^D^	158.5 ± 36.2	75.2 ± 67.9
	Synbiotic B	242.5 ± 42.5	6.3 ± 0.8	0.3 ± 0.1 *^D^	152.7 ± 21.7	67.7 ± 42.6
	Synbiotic C	220.2 ± 30.3	5.9 ± 1.6	0.3 ± 0.1 *^D^	174.0 ± 12.0	54.7 ± 10.3
	Bioplus^®^ YC (D)	220.9 ± 29.3	7.4 ± 3.5	0.7 ± 0.3 *^ABCEK^	153.6 ± 7.9	52.1 ± 6.1
	Cylactin^®^ (E)	217.9 ± 22.8	7.0 ± 2.4	0.4 ± 0.1 *^D^	164.0 ± 16.5	64.2 ± 23.2
	Without (K)	217.1 ± 18.8	7.6 ± 2.7	0.4 ± 0.1 *^D^	166.0 ± 15.5	68.4 ± 24.1
**28 days**	Synbiotic A	261.3 ± 13.7 *^BCD^	3.1 ± 0.9 *^CK^	0.2 ± 0.1	140.2 ± 22.0	27.9 ± 10.8
	Synbiotic B	209.0 ± 22.0 *^AE^	6.8 ± 3.7	0.2 ± 0.1	155.6 ± 15.4	34.0 ± 7.6
	Synbiotic C	207.7 ± 22.4 *^AE^	7.8 ± 3.2 *^A^	0.2 ± 0.1	171.5 ± 49.8	34.2 ± 24.8
	Bioplus^®^ YC (D)	210.1 ± 33.3 *^AE^	5.1 ± 4.4 *^K^	0.3 ± 0.2	135.4 ± 14.8	32.4 ± 5.7
	Cylactin^®^ (E)	268.2 ± 14.7 *^BCD^	4.3 ± 1.0 *^K^	0.3 ± 0.1	147.3 ± 16.2	46.3 ± 4.7
	Without (K)	250.4 ± 16.9	9.9 ± 6.0 *^ADE^	0.3 ± 0.0	156.1 ± 20.1	54.1 ± 14.4
**42 days**	Synbiotic A	170.2 ± 72.6	2.6 ± 0.4	0.4 ± 0.5	135.6 ± 24.0	27.8 ± 7.3
	Synbiotic B	213.8 ± 23.6	2.8 ± 0.4	0.2 ± 0.1 *^D^	146.4 ± 18.8	28.8 ± 7.0
	Synbiotic C	210.2 ± 14.6	2.5 ± 0.8	0.2 ± 0.1 *^DE^	144.3 ± 34.4	27.2 ± 8.9
	Bioplus^®^ YC (D)	215.9 ± 61.2	3.5 ± 1.5	0.6 ± 0.2 *^BCE^	123.9 ± 14.5	29.6 ± 7.8
	Cylactin^®^ (E)	197.5 ± 15.0	4.8 ± 1.5	0.2 ± 0.1 *^D^	143.5 ± 12.6	38.3 ± 13.8
	Without (K)	196.5 ± 15.8	3.8 ± 1.1	0.4 ± 0.2	145.8 ± 20.6	37.0 ± 14.6

**Table 6:** Concentrations of the tested parameters in the blood serum of the experimental chickens. *—statistically significant differences between groups, *p* ≤ 0.05.

Age of Birds	Feed Additives	Total Protein (g/L)	Albumin (g/L)	Globulin (g/L)	A:G ratio (Albumin:Globulin)
**7 days**	Synbiotic A	22.5 ± 3.9	13.2 ± 1.2	9.3 ± 3.1	1.7 ± 1.1
	Synbiotic B	24.0 ± 2.5	14.3 ± 2.4	9.7 ± 2.7	1.6 ± 0.8
	Synbiotic C	24.0 ± 2.8	13.2 ± 1.8	10.8 ± 1.2	1.2 ± 0.1
	Bioplus^®^ YC (D)	23.5 ± 2.1	12.7 ± 0.5	10.8 ± 1.8	1.2 ± 0.2
	Cylactin^®^ (E)	22.2 ± 2.3	12.5 ± 1.5	9.7 ± 2.9	1.4 ± 0.5
	Without (K)	21.7 ± 2.2	11.3 ± 1.0	10.3 ± 1.4	1.1 ± 0.1
**14 days**	Synbiotic A	23.0 ± 2.6	13.3 ± 1.6	9.7 ± 2.3	1.5 ± 0.5
	Synbiotic B	23.0 ± 2.8	12.7 ± 1.2	10.3 ± 1.6	1.2 ± 0.1
	Synbiotic C	23.5 ± 2.1	13.0 ± 1.4	10.5 ± 0.8	1.2 ± 0.1
	Bioplus^®^ YC (D)	23.5 ± 1.2	13.5 ± 0.5	10.0 ± 1.4	1.4 ± 0.2
	Cylactin^®^ (E)	24.2 ± 2.6	13.5 ± 1.5	10.7 ± 1.8	1.3 ± 0.3
	Without (K)	24.3 ± 2.9	13.5 ± 1.4	10.8 ± 2.3	1.3 ± 0.3
**28 days**	Synbiotic A	28.0 ± 4.8	14.8 ± 1.9 *^BCDEK^	13.2 ± 5.5	1.3 ± 0.6
	Synbiotic B	30.2 ± 2.9	16.2 ± 1.5 *^A^	14.0 ± 1.9	1.2 ± 0.1
	Synbiotic C	32.5 ± 12.1 *^EK^	18.2 ± 6.9 *^A^	14.3 ± 5.3	1.3 ± 0.1
	Bioplus^®^ YC (D)	27.7 ± 2.0	15.5 ± 1.0 *^A^	12.2 ± 1.2	1.3 ± 0.1
	Cylactin^®^ (E)	26.3 ± 1.9 *^C^	15.3 ± 1.2 *^A^	11.0 ± 2.5	1.5 ± 0.4
	Without (K)	25.8 ± 2.4 *^C^	15.3 ± 1.0 *^A^	10.5 ± 1.5	1.5 ± 0.2
**42 days**	Synbiotic A	28.2 ± 8.3	13.8 ± 3.9	14.3 ± 4.7	1.0 ± 0.1
	Synbiotic B	33.0 ± 2.1	15.8 ± 3.3	17.2 ± 3.1	1.0 ± 0.3
	Synbiotic C	30.8 ± 2.9	16.7 ± 2.0	14.2 ± 1.8	1.2 ± 0.2
	Bioplus^®^ YC (D)	31.7 ± 2.3	16.2 ± 0.8	15.5 ± 1.5	1.0 ± 0.1
	Cylactin^®^ (E)	30.7 ± 1.9	15.5 ± 0.8	15.2 ± 1.2	1.0 ± 0.0
	Without (K)	29.7 ± 3.7	15.3 ± 1.5	14.3 ± 2.2	1.1 ± 0.1

**Table 7:** Levels of the tested elements in the chicken blood serum. *—statistically significant differences between groups, *p* ≤ 0.05.

Age of Birds	Feed Additives	Ca (mg/dL)	P (mg/dL)	Ca:P Ratio	K (mmol/L)	Na (mmol/L)
**7 days**	Synbiotic A	12.7 ± 1.7 *^CEK^	5.7 ± 1.0	2.3 ± 0.5	5.9 ± 0.9 *^D^	142.8 ± 2.7
	Synbiotic B	11.3 ± 2.4 *^EK^	4.4 ± 1.2 *^EK^	2.8 ± 1.0	4.9 ± 0.5 *^DEK^	151.0 ± 7.1 *^D^
	Synbiotic C	9.4 ± 1.2 *^ADEK^	4.8 ± 0.7	2.0 ± 0.5	5.5 ± 2.1 *^DEK^	141.9 ± 3.9
	Bioplus^®^ YC (D)	13.2 ± 1.3 *^C^	6.4 ± 1.3	2.1 ± 0.3	7.5 ± 0.7 *^ABC^	137.8 ± 3.7 *^B^
	Cylactin^®^ (E)	12.1 ± 2.3 *^ABC^	6.2 ± 0.9 *^B^	2.0 ± 0.4	6.2 ± 0.8 *^BC^	139.4 ± 5.2
	Without (K)	14.9 ± 1.5 *^ABC^	7.0 ± 0.9 *^B^	2.1 ± 0.2	7.2 ± 0.6 *^BC^	141.5 ± 1.8
**14 days**	Synbiotic A	11.8 ± 1.3 ^*CDEK^	5.2 ± 0.8	2.3 ± 0.4	8.0 ± 0.5	140.7 ± 2.3
	Synbiotic B	13.2 ± 2.0 *^CDEK^	5.0 ± 0.9	2.7 ± 0.8	7.1 ± 0.7	141.6 ± 1.4
	Synbiotic C	16.5 ± 1.2 *^AB^	4.5 ± 0.9	3.7 ± 0.8	7.2 ± 0.4	142.1 ± 2.9
	Bioplus^®^ YC (D)	16.4 ± 2.1 *^AB^	6.6 ± 1.9	2.7 ± 1.1	7.9 ± 1.2	146.1 ± 3.6
	Cylactin^®^ (E)	14.8 ± 2.3 *^AB^	5.7 ± 1.2	2.6 ± 0.4	7.6 ± 0.7	147.2 ± 3.2
	Without (K)	15.8 ± 1.1 *^AB^	6.5 ± 1.3	2.5 ± 0.5	7.8 ± 0.8	142.8 ± 10.1
**28 days**	Synbiotic A	10.9 ± 1.0	6.7 ± 1.8	1.7 ± 0.3	5.6 ± 1.2	145.2 ± 3.1
	Synbiotic B	9.7 ± 1.0	7.7 ± 1.4	1.3 ± 0.2	5.4 ± 0.5	146.2 ± 1.9
	Synbiotic C	10.2 ± 2.1	7.7 ± 2.6	1.4 ± 0.5	6.0 ± 0.6 *^EK^	146.6 ± 1.8
	Bioplus^®^ YC (D)	9.7 ± 0.7	8.9 ± 0.5 *^K^	1.1 ± 0.1	6.4 ± 0.4 *^EK^	145.2 ± 1.5
	Cylactin^®^ (E)	8.6 ± 0.8	7.1 ± 0.9	1.2 ± 0.1	4.2 ± 0.2 *^CD^	146.4 ± 4.1
	Without (K)	10.7 ± 0.7	5.7 ± 1.4 *^D^	2.0 ± 0.5	4.1 ± 0.6 *^CD^	150.0 ± 2.3
**42 days**	Synbiotic A	10.3 ± 0.4	7.5 ± 1.0	1.4 ± 0.1	8.3 ± 1.3	131.2 ± 24.3 *^BCDEK^
	Synbiotic B	10.1 ± 0.7	7.0 ± 0.6	1.4 ± 0.1	8.2 ± 1.2	145.0 ± 1.0 *^A^
	Synbiotic C	9.6 ± 0.7	8.1 ± 3.2	1.3 ± 0.3	7.8 ± 1.1	142.5 ± 2.5 *^A^
	Bioplus^®^ YC (D)	9.9 ± 0.9	7.9 ± 0.9	1.3 ± 0.1	8.0 ± 1.4	143.7 ± 1.8 *^A^
	Cylactin^®^ (E)	9.6 ± 0.6	7.6 ± 0.9	1.3 ± 0.2	7.7 ± 0.8	145.0 ± 2.3 *^A^
	Without (K)	9.5 ± 0.5	7.9 ± 0.8	1.2 ± 0.1	7.8 ± 1.2	145.8 ± 2.5 *^A^
